# Severe concentric hypertrophy after cardiac arrest makes support with ECPELLA® impossible

**DOI:** 10.1093/ehjimp/qyae112

**Published:** 2024-10-30

**Authors:** Maria Vidal-Burdeus, Eduard Argudo, Imanol Otaegui-Irureta, Jordi Riera-Del Brio, Aitor Uribarri

**Affiliations:** Cardiology Department, Hospital Universitari Vall d’Hebron, Passeig Vall d’Hebron 119-129, 08035 Barcelona, Spain; Intensive Care Department, Hospital Universitari Vall d’Hebron, Passeig Vall d’Hebron 119-129, 08035 Barcelona, Spain; SODIR Shock Organ Dysfunction and Resuscitation Research Group, Vall d’Hebron Institut de Recerca (VHIR), Passeig Vall d’Hebron 119-129, 08035 Barcelona, Spain; Cardiology Department, Hospital Universitari Vall d’Hebron, Passeig Vall d’Hebron 119-129, 08035 Barcelona, Spain; CIBER CV, Centro de Investigacion Biomedica en Red (CIBER) Instituto Salud Carlos III, Avda. Monforte de Lemos, 3-5 Pabellón 11, 28029 Madrid, Spain; VHIR, Fundació Hospital Universitari Vall d'Hebron - Institut de Recerca, Passeig Vall d'Hebron 119-129, 08035 Barcelona, Spain; Intensive Care Department, Hospital Universitari Vall d’Hebron, Passeig Vall d’Hebron 119-129, 08035 Barcelona, Spain; SODIR Shock Organ Dysfunction and Resuscitation Research Group, Vall d’Hebron Institut de Recerca (VHIR), Passeig Vall d’Hebron 119-129, 08035 Barcelona, Spain; Cardiology Department, Hospital Universitari Vall d’Hebron, Passeig Vall d’Hebron 119-129, 08035 Barcelona, Spain; CIBER CV, Centro de Investigacion Biomedica en Red (CIBER) Instituto Salud Carlos III, Avda. Monforte de Lemos, 3-5 Pabellón 11, 28029 Madrid, Spain; VHIR, Fundació Hospital Universitari Vall d'Hebron - Institut de Recerca, Passeig Vall d'Hebron 119-129, 08035 Barcelona, Spain

**Keywords:** cardiac arrest, ECPELLA, severe concentric hypertrophy

A 56-year-old male patient with no medical history was admitted for refractory cardiac arrest (CA) with ventricular fibrillation as the initial rhythm. Veno-arterial extracorporeal membrane oxygenation cannulation was performed, initiating support 63 min after CA.

Coronary angiography showed occlusion of the proximal anterior descending artery, successfully treated with a drug-eluting stent. Transthoracic echocardiogram revealed a left ventricle with severe concentric hypertrophy and significant systolic dysfunction (*Images A-B).

Due to elevated intra-cavitary pressures and absence of aortic valve (AoV) opening, an Impella-CP® was implanted. The Impella® generated continuous suction events and did not provide adequate flow to the patient (Impella® console with arterial curve without pulsatility and reduction in motor current, Image C) despite being correctly positioned (AoV at the radiopaque marker, **Image D).

The transoesophageal echocardiogram was not available. Therefore, ventriculography was performed. A small left ventricular cavity with severe mitral insufficiency due to anterior leaflet traction by the device was observed (see Supplementary data online, *Video S1*-*S2*). Repositioning of the Impella® was unsuccessful, and it was replaced with an intra-aortic balloon pump (IABP).

Unfortunately, the patient suffered brain herniation and was diagnosed with brain death 24 h after admission.

The use of Impella® alongside VA-ECMO is becoming more common. However, in some circumstances, like our patient, it may not be the most effective approach. After refractory CA, significant myocardial oedema can reduce the size of the left ventricular cavity, impairing Impella® function. In these cases, an IABP may be more appropriate as unloading measures.

**Figure:**
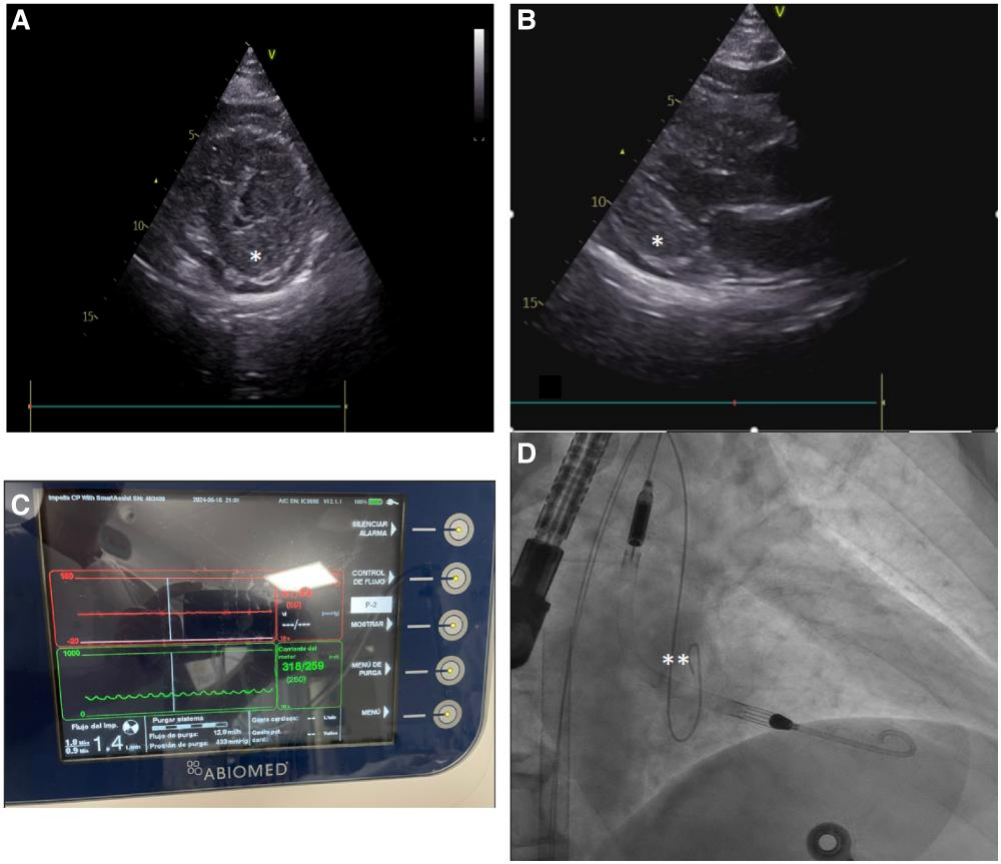


## Supplementary data

Supplementary data are available at *European Heart Journal - Imaging Methods and Practice* online.

## Consent

Informed consent was obtained from the patient's relatives for publication of this case report.

**Funding:** None declared.

## Supplementary Material

qyae112_Supplementary_Data

## Lead author biography

Graduate in Medicine from the Universitat Autonoma de Barcelona. Medical specialist in cardiology at the Joan XXIII University Hospital in Tarragona. Fellowship in cardiovascular critical care at Hospital De la Santa Creu i Sant Pau in Barcelona. Currently attached to the Cardiovascular Critical Care Unit at the Vall d'Hebron University Hospital in Barcelona.

